# Short-term outcomes of robotic- versus laparoscopic-assisted Total Gastrectomy for advanced gastric Cancer: a propensity score matching study

**DOI:** 10.1186/s12885-020-07160-1

**Published:** 2020-07-17

**Authors:** Changdong Yang, Yan Shi, Shaohui Xie, Jun Chen, Yongliang Zhao, Feng Qian, Yingxue Hao, Bo Tang, Peiwu Yu

**Affiliations:** Department of General Surgery, Southwest Hospital, Army Medical University, 30 Gaotanyan Street, Shapingba District, 400038 Chongqing China

**Keywords:** Advanced gastric cancer, Total gastrectomy, Robotic, Laparoscopic, Short-term outcomes

## Abstract

**Background:**

Few studies have been designed to evaluate the short-term outcomes between robotic-assisted total gastrectomy (RATG) and laparoscopy-assisted total gastrectomy (LATG) for advanced gastric cancer (AGC). The purpose of this study was to assess the short-term outcomes of RATG compared with LATG for AGC.

**Methods:**

We retrospectively evaluated 126 and 257 patients who underwent RATG or LATG, respectively. In addition, we performed propensity score matching (PSM) analysis between RATG and LATG for clinicopathological characteristics to reduce bias and compared short-term surgical outcomes.

**Results:**

After PSM, the RATG group had a longer mean operation time (291.14 ± 59.18 vs. 270.34 ± 52.22 min, *p* = 0.003), less intraoperative bleeding (154.37 ± 89.68 vs. 183.77 ± 95.39 ml, *p* = 0.004) and more N2 tier RLNs (9.07 ± 5.34 vs. 7.56 ± 4.50, *p* = 0.016) than the LATG group. Additionally, the total RLNs of the RATG group were almost significantly different compared to that of the LATG group (34.90 ± 13.05 vs. 31.91 ± 12.46, *p* = 0.065). Moreover, no significant differences were found between the two groups in terms of the length of incision, proximal resection margin, distal resection margin, residual disease and postoperative hospital stay. There was no significant difference in the overall complication rate between the RATG and LATG groups after PSM (23.8% vs. 28.6%, *p* = 0.390). Grade II complications accounted for most of the complications in the two cohorts after PSM. The conversion rates were 4.55 and 8.54% in the RATG and LATG groups, respectively, with no significant difference (*p* = 0.145), and the ratio of splenectomy were 1.59 and 0.39% (*p* = 0.253). The mortality rates were 0.8 and 0.4% for the RATG and LATG groups, respectively (*p* = 1.000).

**Conclusion:**

This study demonstrates that RATG is comparable to LATG in terms of short-term surgical outcomes.

## Background

Gastric cancer (GC) is the fifth most common cancer and the third leading cause of cancer-related death worldwide [1]. Its incidence and mortality rates have been steadily declining worldwide since the middle of the 20th century [2, 3]. However, it is notable that the morbidity of esophagogastric junction cancer is increasing in Western and Eastern countries [2–5]. Total gastrectomy (TG) with adequate regional lymphadenectomy is the most common treatment choice for upper GC and includes cancers located in the proximal third of the stomach and esophagogastric junction (EGJ) (Siewert type II and III) or cancers located at the lower two-thirds of the stomach to ensure a tumour-free surgical margin [6–8]. Since Kitano [9] reported laparoscopy-assisted distal gastrectomy in 1994 for the first time, laparoscopy-assisted gastrectomy has been widely used for gastric cancer [10–12]. Despite its technical difficulty, laparoscopy-assisted total gastrectomy (LATG) has been shown to be technically feasible and is superior to open total gastrectomy performed by experienced surgeons in terms of its safety and short-term outcomes [13, 14]. However, the two-dimensional visualization and limited movement of laparoscopic instruments make it difficult to perform lymphadenectomy precisely. Robotic surgical system overcomes those limitations including eliminating the traces of physiologic human tremor and increasing dexterity through its typical internal articulated endoscopic wrist (EndoWrist™ System) for a precise lymphadenectomy with a 3D high-resolution images at the console [15]. In 2002, Hashizume reported robotic-assisted gastrectomy for the first time [16]. Since then, robotic surgery has been demonstrated to obtain similar or even better anatomical and operative conditions compared to the traditional laparoscopic approach during gastric resection [15, 17–21]. However, most of the reported cases were early gastric cancer (EGC) [22, 23], and few studies have retrospectively compared robotic-assisted total gastrectomy (RATG) with LATG for advanced gastric cancer (AGC) [15, 24]. The aim of this study is to evaluate the feasibility and safety of RATG and LATG for AGC using the propensity score matching (PSM) method.

## Methods

### Patients

Patients diagnosed with GC by means of gastroscopy, biopsy and histopathological assessment who underwent total gastrectomy were screened from the prospectively maintained gastric cancer database at the Department of General Surgery, Southwest Hospital, Army Medical University from March 2010 to December 2017. Data from 573 consecutive patients who underwent RATG or LATG for gastric cancer were collected. The inclusion criteria of the study were defined as follows: (1) age between 18 and 80 years old; (2) no preoperative chemotherapy or radiation therapy performed before surgery; (3) depth of invasion confined to pT2, pT3, or pT4a; (4) no distant metastasis or invasion to adjacent organs; (5) receiving LATG or RATG with D2 lymphadenectomy. Patients who underwent RATG were matched to those who underwent LATG at a 1:1 ratio by using a propensity score matching (PSM) method to reduce the effect of bias due to the imbalanced clinicopathological features of the two groups. The matched variables included age, sex, body mass index (BMI), American Society of Anesthesiologists (ASA) grade, T stage, N stage, tumour-node-metastasis classification (TNM), tumour size, tumour location, Borrmann type, differentiation and comorbidities. Postoperative complications were recorded and classified according to the Clavien-Dindo classification system [25, 26]. Pathological and clinical staging were determined based on the AJCC Cancer Staging Manual (Eighth Edition) [27].

### Operation procedures

All patients underwent standard radical total gastrectomy with D2 lymphadenectomy according to the Guidelines of the Japanese Gastric Cancer Association [7, 28]. The da Vinci Surgical System (Intuitive Surgical, Inc., Sunnyvale, CA) was used as the robotic tool for all patients in the robotic group. During RATG, five surgical ports were inserted in the upper abdomen as we previously described [17]. The details of the gastrectomy and lymph node dissections during the RATG procedures did not differ from those during the LATG procedures except for the use of the articulating robotic instruments. After finishing the lymph node (LN) dissection, the robotic arms were undocked and withdrawn. We conducted Roux-en-Y reconstruction to rebuild the digestive tract in both the RATG and LATG surgeries, mostly through a 6–8 cm upper abdominal incision, as we previously described [17]. When conducting the esophagojejunostomy, the esophagus was transected with an anvil in it, and then the Roux-en-Y limb was brought up to complete an esophagojejunostomy using a 25-mm circular stapler, while the jejunal stump was closed and side-to-side jejunojejunostomy was established using an endoscopic linear stapler [17]. The decision to reinforce the anastomoses or the duodenal stump depended on the operators’ judgement during surgeries, and two drainage tubes were placed under the liver and beside the spleen. All patients were informed of the advantages and disadvantages of RATG and LATG, and an informed consent form was signed before surgery by the patients themselves or their legal representatives. The surgeries were performed by five experienced surgeons who received robotic surgery certification and had performed robotic surgery (RG) with D2 lymphadenectomy in more than 30 cases. RATG and LATG were compared by evaluating the surgical performance and postoperative short-term clinical outcomes, including the operation time, estimated blood loss, proximal resection margin, distal resection margin, number of retrieved lymph nodes (RLNs), postoperative complications and length of postoperative hospital stay.

### Statistical analysis

SPSS version 22.0 for Windows (IBM Corp., Armonk, NY) was used for statistical analysis. R version 3.5.2 for Windows was used for PSM by using the MatchIt package. The independent sample *t* test, Mann–Whitney test and chi-square test were used for continuous variables or categorical variables. Continuous variables are presented as the mean ± standard deviation (SD). A value of *p <* 0.05 was considered statistically significant, and all *p* values were two-sided.

## Results

### Clinicopathological characteristics

**Fig. 1:**
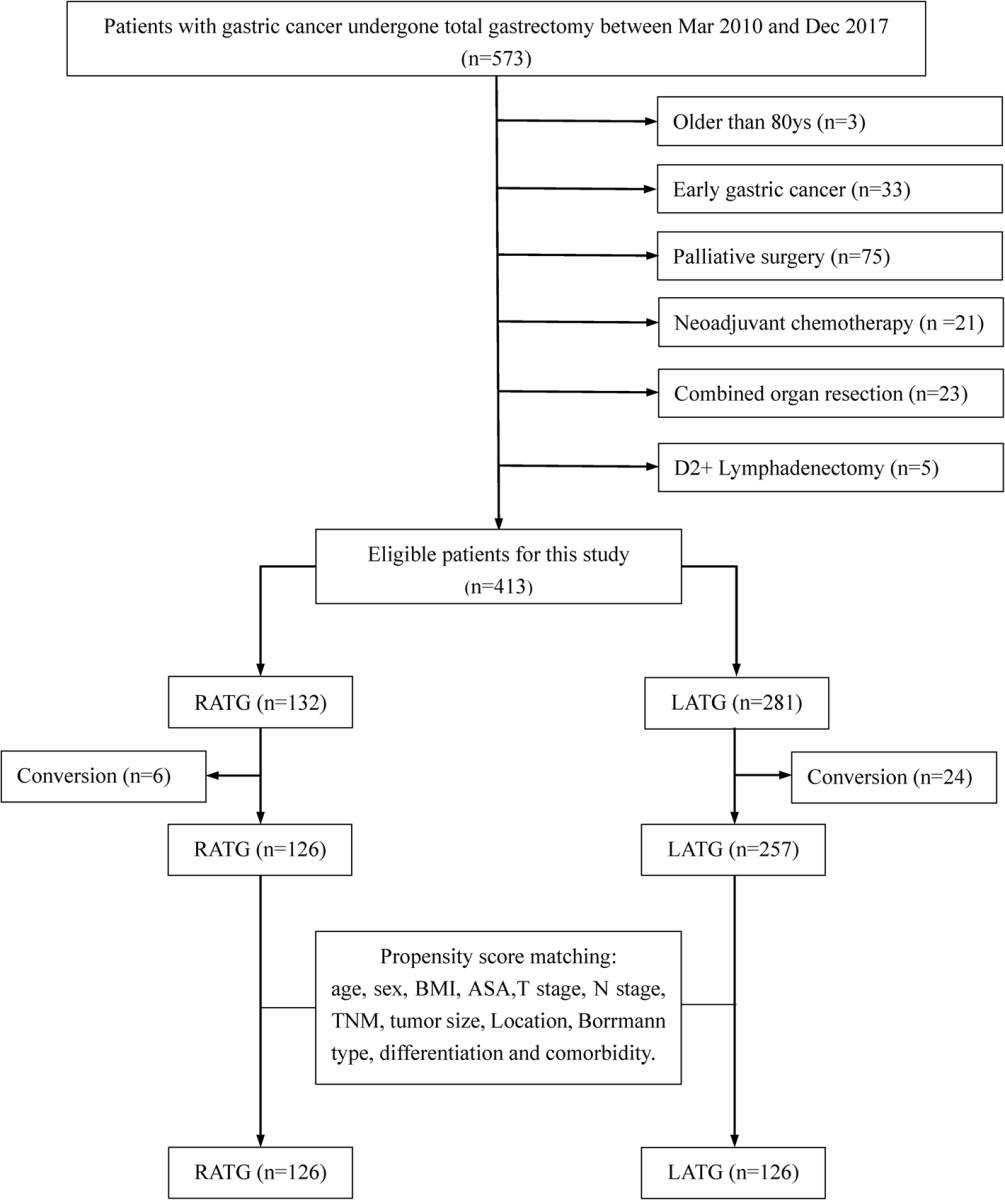
Population flowchart

A total of 160 patients were excluded for the following reasons: patients were over 80 years old (*n* = 3), had early gastric cancer (*n* = 33), received palliative surgery (*n* = 75), received neoadjuvant chemotherapy before surgery (*n* = 21), underwent combined organ resection (*n* = 23), underwent D2+ lymphadenectomy (*n* = 5). The statistical analyses were performed in the remaining 413 patients undergoing radical total gastrectomy, of whom 132 underwent RATG and 281 underwent LATG (Fig. 1). Finally, the study cohort comprised 126 patients who underwent RATG and 126 matched LATG patients after PSM. The patients’ clinicopathological characteristics before and after PSM are summarized in Table 1. The patients in the two groups before PSM were generally matched with no significant differences (*p* > 0.05) in age, sex, BMI, ASA grade, Borrmann type, N stage, TNM stage, or medical comorbidities (such as diabetes, hypertension, heart disease and contagious disease), except T stage, tumour differentiation and abdominal surgery history (*p* < 0.05). However, those biases were reduced after PSM, and the clinicopathological characteristics were better matched between the two groups.

**Table 1:** Clinicopathological characteristics

Variables	All Patients			Patients after PSM		
	RATG(*n* = 126)	LATG(*n* = 257)	*p*	RATG(*n* = 126)	LATG(*n* = 126)	*p*
Age, year (mean ± SD)	60.33 ± 8.94	58.26 ± 10.41	0.051	60.33 ± 8.94	60.78 ± 9.05	0.690
Sex (male/female)	105/21	197/60	0.133	105/21	100/26	0.419
Height, cm (mean ± SD)	163.52 ± 6.58	162.74 ± 7.25	0.304	163.52 ± 6.58	162.79 ± 7.91	0.422
Weight, Kg (mean ± SD)	59.21 ± 8.37	59.63 ± 9.46	0.667	59.21 ± 8.37	58.84 ± 9.70	0.745
BMI, Kg/m^2^(mean ± SD)	22.10 ± 2.48	22.46 ± 2.93	0.200	22.10 ± 2.48	22.13 ± 2.84	0.929
ASA (I/II/III)	87/36/3	185/68/4	0.529	87/36/3	94/30/2	0.320
Tumor size, cm (mean ± SD)	4.62 ± 2.22	4.55 ± 2.28	0.759	4.62 ± 2.22	4.40 ± 2.35	0.446
Tumor location			0.398			0.661
Siewert type II	28	61		28	28	
Siewert type III	30	69		30	33	
Body	68	127		68	65	
Borrmann type			0.076			0.785
I/II/III/IV	3/11/100/12	11/27/207/12		3/11/100/12	2/10/106/8	
Depth of infiltration (T)			0.023			0.796
T2	7	36		7	9	
T3	2	3		2	1	
T4a	117	218		117	116	
Nodal status (N)			0.483			0.841
N0	29	64		29	30	
N1	29	45		29	25	
N2	27	43		27	25	
N3a	21	61		21	29	
N3b	20	44		20	17	
TNM stage			0.814			0.803
IB	3	23		3	3	
IIA	6	7		6	0	
IIB	24	44		24	27	
IIIA	52	81		52	45	
IIIB	21	58		21	31	
IIIC	20	44		20	10	
Differentiation			0.006			0.534
G1/G2/G3	0/28/98	1/92/164		0/28/98	0/24/102	
Comorbidities (0/1/2/3)	91/23/6/6	174/52/25/6	0.385	91/23/6/6	91/22/11/2	0.983
Abdominal surgery history (Y/N)	5/121	26/231	0.038	5/121	13/113	0.050

*RATG* Robotic-assisted total gastrectomy, *LATG* Laparoscopic-assisted total gastrectomy, *PSM* Propensity Score Matching, *SD* Standard Deviation, *BMI* body mass index, *ASA* American Society of Anesthesiologists, *TNM* tumor-node-metastasis, *G1/G2/G3* High/Middle/Low or Mucus differentiation, *Comorbidities (0/1/2/3)* no/one/two/three comorbidities, *Y* Yes, *N* No.

### Short-term surgical outcomes of the cohorts

The postoperative clinical outcomes before and after PSM are detailed in Table 2. Before PSM, the RATG group had a longer mean operation time (291.14 ± 59.18 vs. 270.27 ± 49.41 min, *p* = 0.003), less intraoperative bleeding (154.37 ± 89.68 vs. 175.19 ± 105.44 ml, *p* = 0.028), more total RLNs (34.90 ± 13.05 vs. 32.02 ± 12.41, *p* = 0.037), and more N2 tier RLNs (9.07 ± 5.34 vs. 7.61 ± 4.57, *p* = 0.007) than the LATG group. After PSM, the RATG group still had a longer mean operation time (291.14 ± 59.18 vs. 270.34 ± 52.22 min, *p* = 0.003), less intraoperative bleeding (154.37 ± 89.68 vs. 183.77 ± 95.39 ml, *p* = 0.004) and more N2 tier RLNs (9.07 ± 5.34 vs. 7.56 ± 4.50, *p* = 0.016) than the LATG group. Additionally, the total RLNs of the RATG group were almost significantly different compared to that of the LATG group (34.90 ± 13.05 vs. 31.91 ± 12.46, *p* = 0.065). Moreover, no significant differences were found between the two groups in terms of the length of incision, proximal resection margin, distal resection margin, residual disease and postoperative hospital stay.

**Table 2:** Comparison of surgical outcomes and postoperative recovery

Variables	All Patients			Patients after PSM		
	RATG(*n* = 126)	LATG(*n* = 257)	*p*	RATG(*n* = 126)	LATG(*n* = 126)	*p*
Operation time, min (mean ± SD)	291.14 ± 59.18	270.27 ± 49.41	0.003	291.14 ± 59.18	270.34 ± 52.22	0.003
Bleeding, ml (mean ± SD)	154.37 ± 89.68	175.19 ± 105.44	0.028	154.37 ± 89.68	183.77 ± 95.39	0.004
Retrieved lymph nodes (mean ± SD)	34.90 ± 13.05	32.02 ± 12.41	0.037	34.90 ± 13.05	31.91 ± 12.46	0.065
N1 tier (mean ± SD)	25.83 ± 10.68	24.41 ± 10.09	0.206	25.83 ± 10.68	24.36 ± 10.00	0.261
N2 tier (mean ± SD)	9.07 ± 5.34	7.61 ± 4.57	0.007	9.07 ± 5.34	7.56 ± 4.50	0.016
Length of incision, cm (mean ± SD)	6.32 ± 1.58	6.34 ± 1.75	0.546	6.32 ± 1.58	6.46 ± 1.87	0.914
Proximal margin, cm (mean ± SD)	3.55 ± 1.69	3.67 ± 1.43	0.488	3.55 ± 1.69	3.67 ± 1.53	0.553
Distal margin, cm (mean ± SD)	7.14 ± 3.68	7.59 ± 3.79	0.275	7.14 ± 3.68	7.72 ± 3.83	0.225
R0/R1	116/10	244/13	0.265	116/10	118/8	0.625
Postoperative hospital stay, d (mean ± SD)	9.62 ± 2.86	9.93 ± 4.00	0.430	9.62 ± 2.86	9.86 ± 4.31	0.606

*RATG* Robotic-assisted total gastrectomy, *LATG* Laparoscopic-assisted total gastrectomy, *PSM* Propensity Score Matching, *SD* Standard Deviation, *R* Residual disease(R classification)

Six patients underwent conversion to laparotomy in the robotic group and 24 in the laparoscopic group (4.55% vs. 8.54%, *p* = 0.145). In the robotic group, 2 patients encountered uncontrollable bleeding, 2 caused by tight adhesion and 2 had the left gastric artery surrounded by lymph nodes. In the laparoscopic group, 13 patients had tight adhesion, 4 had the left gastric artery surrounded by lymph nodes, 2 caused by enlarged lymph nodes, 1 caused by the tumour surrounding the artery, 2 caused by a giant tumour, 1 encountered bleeding of a short gastric vessel, and 1 encountered mechanical failure of the stapler. Furthermore, two patients underwent splenectomy in the robotic group, and one underwent splenectomy in the laparoscopic group because of the tight adhesion of the spleen hilum (1.59% vs. 0.39%, *p* = 0.253).

The postoperative complications before and after PSM are shown in Table 3. There was no significant difference in the overall complication rate between the RATG and LATG groups before PSM (23.8% vs. 29.2%, *p* = 0.268) and after PSM (23.8% vs. 28.6%, *p* = 0.390). Grade II complications accounted for most of the complications in the two cohorts both before and after PSM. Moreover, no significant differences were noted in the major complications (Clavien-Dindo grade ≥ IIIa) among all complications between the two cohorts before PSM (5.6% vs. 8.2%, *p* = 0.356) and after PSM (5.6% vs. 5.6%, *p* = 1.000). One patient in the RATG died of MODS after anastomotic leakage who received a second surgical procedure. One patient in the LATG died of MODS after pulmonary failure. The mortality rates were 0.8 and 0.4% for the RATG and LATG groups, respectively (*p* = 1.000).

**Table 3:** Postoperative morbidity and mortality

Variables	All Patients			Patients after PSM		
	RATG(n = 126)	LATG(n = 257)	*p*	RATG(n = 126)	LATG(n = 126)	*p*
Present/absent	30/96 (23.8%)	75/182 (29.2%)	0.268	30/96 (23.8%)	36/90 (28.6%)	0.390
Clavien-Dindo Classification						
I	3 (2.4%)	11 (4.3%)	0.352	3 (2.4%)	7 (5.6%)	0.197
Wound problem	2	5		2	2	
Fever	1	5		1	3	
Cardiac dysfunction	0	2		0	2	
Diarrhea	0	2		0	1	
Chylous leakage	0	1		0	1	
II	20 (15.9%)	43 (16.7%)	0.831	20 (15.9%)	22 (17.5%)	0.735
Fever	5	4		5	3	
Wound infection	0	1		0	1	
Intra-abdominal infection	2	7		2	4	
Intestinal obstruction	1	0		1	0	
Catheter infections	4	1		4	1	
Pulmonary infection	8	21		8	11	
Pulmonary atelectasis	0	4		0	0	
Pleural effusion	3	10		3	4	
Anastomotic leakage	2	6		2	1	
Anastomotic stenosis	2	1		2	0	
Intra-abdominal bleeding	1	2		1	2	
Duodenal stump leakage	0	2		0	1	
Cardiac dysfunction	0	2		0	1	
IIIa	2 (1.6%)	9 (3.5%)	0.466	2 (1.6%)	3 (2.4%)	1.000
Wound problem	0	2		0	1	
Duodenal stump leakage	1	0		1	0	
Anastomotic leakage	0	3		0	1	
Pleural effusion	1	6		1	2	
Pyothorax	0	1		0	0	
Intra-abdominal infection	0	5		0	2	
IIIb	2 (1.6%)	4 (1.6%)	1.000	2 (1.6%)	0 (0%)	0.478
Intra-abdominal bleeding	1	1		1	0	
Anastomotic bleeding	0	1		0	0	
Duodenal stump leakage	0	1		0	0	
Anastomotic leakage	1	1		1	0	
IVa	2 (1.6%)	4 (1.6%)	1.000	2 (1.6%)	2 (1.6%)	1.000
Respiratory failure	2	3		2	1	
Cardiac failure	0	1		0	1	
IVb	0 (0%)	3 (1.2%)	0.554	0 (0%)	1 (0.8%)	1.000
MODS	0	3		0	1	
V	1 (0.8%)	1 (0.4%)	0.550	1 (0.8%)	1 (0.8%)	1.000
Clavien-Dindo grade ≥ IIIa	7 (5.6%)	21 (8.2%)	0.356	7 (5.6%)	7 (5.6%)	1.000
Mortality	1 (0.8%)	1 (0.4%)	0.550	1 (0.8%)	1 (0.8%)	1.000

*RATG* Robotic-assisted total gastrectomy, *LATG* Laparoscopic-assisted total gastrectomy, *PSM* Propensity Score Matching, *MODS* Multiple Organ Dysfunction Syndrome

### Stratified analysis of different related factors

We evaluated the surgical outcomes of the patients according to different related factors, including tumour location, tumour size and age. The surgical outcomes of subgroup analyses are summarized in Tables 4–6*.* Subgroup analysis of tumour location *(*Table 4) suggested that the RATG group had less blood loss than the LATG group when the tumour was located at the esophagogastric junction, while there was no significant difference between the two groups when the tumour was located at the non-esophagogastric junction. Subgroup analysis of tumour size measured by resection specimen suggested that the RATG group had a longer operation time and more N2 tier RLNs compared with the LATG group in patients with tumour sizes smaller than 5 cm, while there was no significant difference between the two groups in patients with tumour sizes larger than 5 cm (Table 5*)*. RATG had less intraoperative bleeding and more N2 tier RLNs compared with the LATG group in patients with age younger than 65 years old, while there was no significant difference between them in patients older than 65 years old (Table 6).

**Table 4:** Comparison of the 2 surgical methods between different tumor location after PSM

	Location EGJ			Location non-EGJ		
	RATG(*n* = 58)	LATG(*n* = 61)	*p*	RATG(*n* = 68)	LATG(*n* = 65)	*p*
Age	61.64 ± 8.57	62.41 ± 7.80	0.608	59.21 ± 9.16	59.25 ± 9.89	0.981
Sex (male/female)	50/8	51/10	0.692	55/13	49/16	0.443
BMI (kg/m^2^)	22.90 ± 2.38	22.45 ± 2.82	0.344	21.41 ± 2.37	21.82 ± 2.84	0.359
Tumor size (cm)	3.66 ± 1.53	4.10 ± 1.51	0.121	5.44 ± 2.39	4.69 ± 2.91	0.106
TNM (IB/IIA/IIB/IIIA/IIIB/IIIC)	2/1/12/27/11/5	1/1/10/29/12/8	0.350	1/5/12/25/10/15	7/0/12/21/16/9	0.611
Comorbidities (present/absent)	18/40	11/50	0.099	17/51	24/41	0.137
Operation time (min)	287.98 ± 51.97	273.07 ± 49.62	0.113	293.84 ± 64.95	267.78 ± 54.80	0.014
Estimated blood loss (ml)	134.66 ± 58.83	173.93 ± 89.41	0.011	171.18 ± 106.95	193.00 ± 100.49	0.085
No. of N2 tier	8.79 ± 4.86	7.43 ± 3.84	0.091	9.31 ± 5.75	7.68 ± 5.07	0.085
No. of Retrieved lymph nodes	35.43 ± 13.38	33.36 ± 11.68	0.184	34.44 ± 12.84	31.49 ± 13.23	0.194
R0/R1	52/6	56/5	0.686	64/4	62/3	1.000
Proximal margin (cm)	2.12 ± 0.99	2.58 ± 1.08	0.013	4.77 ± 1.11	4.69 ± 1.14	0.682
Postoperative complication (%)	18 (31.0)	15 (24.6)	0.433	12 (17.6)	17 (26.2)	0.235
Clavien-Dindo grade ≥ IIIa (%)	5 (8.6)	1 (1.6)	0.187	2 (2.9)	6 (9.2)	0.246
Postoperative hospital stay (d)	9.90 ± 2.77	9.31 ± 1.85	0.176	9.38 ± 2.93	10.37 ± 5.71	0.914

*RATG* Robotic-assisted total gastrectomy, *LATG* Laparoscopic-assisted total gastrectomy, *BMI* body mass index, *TNM* tumor-node-metastasis, *EGJ* esophagogastric junction, *R* Residual disease(R classification)

**Table 5:** Comparison of the 2 surgical methods between different tumor size after PSM

	Size≥5 cm			Size< 5 cm		
	RATG(*n* = 56)	LATG(*n* = 43)	*p*	RATG(*n* = 70)	LATG(*n* = 83)	*p*
Age	61.77 ± 8.23	60.47 ± 8.09	0.433	59.17 ± 9.37	60.94 ± 9.55	0.251
Sex (male/female)	45/11	34/9	0.874	60/10	66/17	0.317
BMI (kg/m^2^)	21.74 ± 2.34	22.52 ± 3.20	0.218	22.38 ± 2.57	21.92 ± 2.62	0.277
Tumor location (non-EGJ/EGJ)	39/17	23/20	0.100	29/41	41/42	0.324
TNM (IB/IIA/IIB/IIIA/IIIB/IIIC)	3/2/8/20/11/12	1/0/8/17/9/8	0.959	0/4/16/32/10/8	7/1/14/33/19/9	0.950
Comorbidities (present/absent)	17/39	13/30	0.989	18/52	22/61	0.912
Operation time (min)	287.46 ± 56.87	278.33 ± 55.51	0.425	294.09 ± 61.20	266.20 ± 50.27	0.002
Estimated blood loss (ml)	159.82 ± 75.14	198.95 ± 110.76	0.132	150.00 ± 100.13	175.90 ± 86.03	0.087
No. of N2 tier	8.64 ± 4.63	8.14 ± 4.78	0.599	9.41 ± 5.86	7.25 ± 4.35	0.010
No. of Retrieved lymph nodes	36.70 ± 13.18	33.14 ± 11.66	0.165	33.46 ± 12.86	31.28 ± 12.88	0.298
R0/R1	53/3	40/3	1.000	63/7	78/5	0.362
Proximal margin (cm)	3.96 ± 1.62	3.85 ± 1.37	0.708	3.21 ± 1.69	3.57 ± 1.61	0.179
Postoperative complication (%)	15 (26.8)	16 (37.2)	0.268	15 (21.4)	20 (24.1)	0.696
Clavien-Dindo grade ≥ IIIa(%)	2 (3.6)	4 (9.3)	0.447	5 (7.1)	3 (3.6)	0.540
Postoperative hospital stay (d)	9.61 ± 1.99	10.58 ± 5.13	0.951	9.63 ± 3.41	9.48 ± 3.80	0.804

*RATG* Robotic-assisted total gastrectomy, *LATG* Laparoscopic-assisted total gastrectomy, *BMI* body mass index, *TNM* tumor-node-metastasis, *EGJ* esophagogastric junction, *R* Residual disease(R classification)

**Table 6:** Comparison of the 2 surgical methods between different age after PSM

	Age ≥ 65ys			Age < 65ys		
	RATG(*n* = 47)	LATG(*n* = 44)	*p*	RATG(*n* = 79)	LATG(*n* = 82)	*p*
Sex (male/female)	42/5	37/7	0.458	63/16	63/19	0.654
BMI (kg/m^2^)	22.54 ± 2.69	21.69 ± 2.68	0.137	21.83 ± 2.32	22.36 ± 2.90	0.214
Tumor location (non-EGJ/EGJ)	20/27	22/22	0.476	48/31	43/39	0.287
Tumor size (cm)	4.39 ± 2.05	4.48 ± 2.70	0.868	4.76 ± 2.31	4.36 ± 2.16	0.263
TNM (IB/IIA/IIB/IIIA/IIIB/IIIC)	2/3/4/17/11/10	4/1/6/15/11/7	0.503	1/3/20/35/10/10	4/0/16/35/17/10	0.340
Comorbidities (present/absent)	14/33	18/26	0.267	21/58	17/65	0.382
Operation time (min)	291.70 ± 71.98	259.98 ± 49.99	0.017	290.81 ± 50.55	275.90 ± 52.83	0.069
Estimated blood loss (ml)	161.81 ± 94.15	174.20 ± 90.68	0.524	149.94 ± 87.23	188.90 ± 97.98	0.037
No. of N2 tier	9.19 ± 6.45	7.41 ± 4.18	0.124	9.00 ± 4.61	7.63 ± 4.69	0.012
No. of Retrieved lymph nodes	34.79 ± 13.33	33.02 ± 12.29	0.514	34.96 ± 12.96	31.32 ± 12.59	0.072
R0/R1	42/5	39/5	1.000	72/5	79/3	0.650
Proximal margin (cm)	3.22 ± 1.57	3.61 ± 1.44	0.221	3.74 ± 1.74	3.70 ± 1.59	0.870
Postoperative complication (%)	14 (29.8)	14 (31.8)	0.834	16 (20.3)	22 (26.8)	0.326
Clavien-Dindo grade ≥ IIIa (%)	3 (6.4)	1 (2.3)	0.657	4 (5.1)	6 (7.3)	0.554
Postoperative hospital stay (d)	9.79 ± 2.81	10.34 ± 4.98	0.512	9.52 ± 2.92	9.60 ± 3.92	0.885

*RATG* Robotic-assisted total gastrectomy, *LATG* Laparoscopic-assisted total gastrectomy, *BMI* body mass index, *TNM* tumor-node-metastasis, *EGJ* esophagogastric junction, *R* Residual disease(R classification)

## Discussion

It is well known that total gastrectomy combined with complete D2 lymphadenectomy and esophagojejunostomy is a technically difficult procedure compared to distal gastrectomy to dissect more lymph nodes [12]. Nonetheless, we described our experience with LATG in the treatment of AGC in 2013, which indicated that LATG was a feasible and safe alternative to standard open gastric resection with similar short-term and long-term results [29]. In regard to RATG, Yoon et al. and Son et al. both reported comparable short-term surgical and oncologic outcomes between RATG and LATG, yet EGC patients accounted for a large percentage of the population in their studies [22, 23]. Ye’s study, which included a total of 205 patients with AGC who underwent RATG or LATG, reported that RATG had a longer operation time, more RLNs, and less operative blood loss and volume of abdominal drainage compared to LATG, and the complication rate was comparable (7.5% vs. 9.1%, *p* = 0.915, 24]. To the best of our knowledge, our study is the first to report the short-term outcomes of RATG compared with LATG for AGC using the PSM method to reduce bias.

Generally, robotic gastrectomy is known to have some advantages over laparoscopic surgery in reducing perioperative bleeding [17, 24, 30]. In our study, we also concluded that robotic surgery can reduce intraoperative bleeding compared to laparoscopic surgery after PSM (154.37 ± 89.68 vs. 183.77 ± 95.39 ml, *p* = 0.004). Although the mean difference of approximately 30 mL of blood loss between the two minimally invasive groups may not provide much clinical benefit for every individual patient, this may show that the robot can operate more accurately to reduce bleeding. However, the present study demonstrated that the operative time of RATG was significantly longer than that of LATG after PSM, which was consistent with the findings of previous studies [22–24]. The docking time of robot arms, the time for arm change during clipping, and the lack of experience of the assistants may explain the longer operative time [22]. The docking time of robotic surgeries was between 20 and 60 min, as reported in a meta-analysis [31]. Since all of our surgeons had performed robotic surgery (RG) for more than 30 cases, the docking time mainly accounted for the prolonged operating time. Hence, the extra time spent in our study (approximately 20 min) for robotic surgery could be acceptable, as docking time was inevitable.

D2 lymphadenectomy is an indispensable process for the application of minimally invasive surgery for AGC [32]. The dissection of the N2 area is the most crucial part of lymphadenectomy. It has been reported that robotic surgery could retrieve more dissected lymph nodes, especially in the technically demanding N2 area, especially in the suprapancreatic area and splenic vessels [33]. In addition, Son et al. found that robotic spleen-preserving total gastrectomy could retrieve more LNs around splenic vessels and the hilum than laparoscopy, and they even compared each group and their metastases [23]. At the same time, the subgroup analysis of a meta-analysis revealed that the number of RLNs of RG was significantly higher than that of LG (*p* = 0.03, 31]. Our study shown that RATG can retrieve more N2 tier RLNs (*p* = 0.007 vs. *p* = 0.016) than LATG both before and after PSM. Nevertheless, the difference in RLNs between the two methods was not clinically significant after PSM. Moreover, the study by Shen et al., which included 23 robotic and 75 laparoscopic total gastrectomy procedures, reported that the RAG and LAG groups had no significant difference in the number of harvested lymph nodes [30]. Li et al. found in their stratified analysis of 92 patients after PSM that the average number of RLNs was not significantly different between robotic and laparoscopic total gastrectomy (30.6 vs. 32.0, *p* = 0.406, 34]. Therefore, it is still controversial whether robotic total gastrectomy can retrieve more lymph nodes. Thus further studies of robotic total gastrectomy, especially RCTs, should be conducted to focus on this issue.

Postoperative complications are an important factor to evaluate the safety and feasibility of a surgical procedure. We evaluated postoperative complications according to the Clavien-Dindo classification system, which is applicable in most parts of the world [25]. Previous studies have proven that the complication rate of laparoscopic total gastrectomy varies from 9.1 to 34.6% [14, 22–24, 34, 35]. In the current study, the complication rate of the RATG group was not significantly different from that of the LATG group before PSM (23.8% vs. 29.2%, *p* = 0.268) and after PSM (23.8% vs. 28.6%, *p* = 0.390). Not surprisingly, pulmonary complications obviously accounted for most of the complications in our study. Upper abdominal surgery combined with pneumoperitoneum and postoperative pain affect the activity of the diaphragm and lead to micro-atelectasis, which in turn causes pulmonary dysfunction. More importantly, total gastrectomy was an independent risk factor for pulmonary complications [36]. Moreover, anastomosis complications were considered to be one of the most serious complications after TG and result in poorer quality of life, prolonged hospital stay, and increased surgery-related costs and mortality [37]. The Japanese National Clinical Database (NCD) of digestive surgery reported that the incidence of anastomotic leakage after total gastrectomy was 4.4% (881 of 20,011) in 2011 [38]. Of the 383 patients included in the analysis, 6 patients in the RATG group and 10 in the LATG group encountered anastomosis-related complications (4.76% vs. 3.89%, *p* = 0.689). The ratio of anastomosis-related complications in the present study was similar with that in previous studies.

Since total gastrectomy was the most common treatment choice for upper gastric cancer, which includes tumours in the proximal third of the stomach and EGJ [6–8], we conducted subgroup analysis according to tumour location. RATG for tumours located at the EGJ showed less intraoperative bleeding and comparable surgical outcomes compared to LATG. As we have mentioned the merits of robot, RG can manage the narrow anatomical fields such as the fundus of the stomach and esophageal hiatus more easily than LG, just as it can overcome the limitations of laparoscopic surgery in the pelvis during rectal surgery [39]. Despite not achieving much statistical significance, RATG have some advantages in dealing with EGJ cancer compared with LATG in our view combined with our limited surgical experience.

However, this study has several limitations. First, the results were based on a retrospective analysis from a single-clinic institution. Second, the present study lacks a detailed comparative analysis of the cost-effectiveness and gastrointestinal function recovery index between robotic and laparoscopic gastric surgery. Third, although the five surgeons who performed the surgeries received robotic surgery certification and were experienced in both minimally invasive surgeries, different surgeons can still cause some bias and further influence the results. Despite this study having some limitations, our findings provide evidence for minimally invasive surgery of total gastrectomy for AGC. Further well-designed studies, especially RCTs or prospective trials, are needed to assess the impact of RATG and LATG.

## Conclusion

This retrospective study demonstrates that RATG is comparable to LATG in terms of short-term surgical outcomes. With longer operation time, less estimated blood loss, more N2 tier RLNs and similar complication rate after PSM, RATG is a safe, reliable and promising approach compared with LATG for the treatment of AGC. Well-designed and randomized controlled trials are needed to further compare RATG with LATG.

## Data Availability

The datasets used and analysed during the current study are available from the corresponding author upon reasonable request.

## Abbreviations

AGC: Advanced gastric cancer
RATG: Robotic-assisted total gastrectomy
LATG: Laparoscopy-assisted total gastrectomy
PSM: Propensity score matching
RLNs: Retrieved lymph nodes
GC: Gastric cancer
TG: Total gastrectomy
LG: Laparoscopy gastrectomy
EGJ: Esophagogastric junction
EGC: Early gastric cancer
BMI: Body mass index
ASA: American Society of Anesthesiologists grade
TNM: Tumor-Node-Metastasis classification
AJCC: American Joint Committee on Cancer
LN: Lymph node
RG: Robotic gastrectomy
SD: Standard deviation
NCD: National Clinical Database
